# The Impact of Cast Walker Design on Metabolic Costs of Walking and Perceived Exertion

**DOI:** 10.3390/diabetology6090098

**Published:** 2025-09-09

**Authors:** Emily Standage, Dylan Tookey, Uchechukwu Ukachukwu, Marco Avalos, Ryan T. Crews, Noah J. Rosenblatt

**Affiliations:** 1 Chicago Medical School, Rosalind Franklin University of Medicine and Science, North Chicago, IL 60024, USA; 2 Center for Lower Extremity Ambulatory Research (CLEAR), Dr. William M. Scholl College of Podiatric Medicine, Rosalind Franklin University of Medicine and Science, North Chicago, IL 60024, USA

**Keywords:** adherence, foot ulcer, diabetes, gait, energetics, biomechanics, strut height, limb length discrepancy

## Abstract

**Background/Objectives:**

Cast walkers (CWs) are often prescribed to offload diabetic foot ulcers (DFUs). However, their mass, the degree of ankle immobilization and the limb length discrepancy they induce may increase the energetic demands of walking, contributing to lower adherence and poorer healing. The purpose of this study was to evaluate the effects of different commercially available CW options on the metabolic costs and perceived exertion of walking, and on related spatiotemporal kinematics, in healthy young participants as an initial step to understanding factors that impact adherence in patients with DFUs.

**Methods:**

Participants walked on an instrumented treadmill at a standardized speed for six minutes under five footwear conditions: (1) athletic shoes only (control); (2) ankle-high CW on the dominant limb with athletic shoe on the contralateral limb; (3) condition two with an external lift on the athletic shoe; (4 and 5) conditions two and three with a knee-high CW. Condition 1 was performed first, after which the CW conditions were randomized. During all conditions, a portable calorimeter recorded gas exchange on a breath-by-breath basis. The metabolic cost of transport (MCoT) was quantified as the mean oxygen consumed per meter walked per kilogram body mass, after accounting for standing. After walking, participants reported perceived exertion using the Borg Rating of Perceived Exertion scale (RPE). From the treadmill data, we extracted the mean step width (SW) as well as absolute values for symmetry indices (SIs) for step length (SL) and step time (ST), all of which have associations with MCoT. For each outcome, linear mixed models compared each CW condition with the control and tested for effects of CW height (ankle-high vs. knee-high) and of the lift.

**Results:**

A total of 14 healthy young adults without diabetes participated. MCoT, RPE and SW were significantly higher for all CW conditions compared to the control, with less consistent results for asymmetry measures. MCoT was not significantly different across CW height or lift condition although an unexpected interaction between limb and CW height n was observed; MCoT was lower in the knee-high CW with vs. without a lift but did not change in the ankle-high CW based on lift status. Similarly, neither SW nor SIs changed in expected fashions across conditions. In contrast, RPE was significantly lower using the ankle- vs. knee-high CW and when using a lift vs no lift, with no significant interaction.

**Conclusions:**

Although metabolic costs were unaffected by CW design changes, which may reflect the absence of anticipated changes in kinematics that impact MCoT, perceived exertion was reduced through such changes. Unanticipated biomechanical changes may reflect a complex interaction among a number of competing factors that dictate behavior and MCoT. The differing results in perception of exertion and metabolic costs might be due to participants’ perceived exertion being sensitive to the collective impact of interacting biomechanical factors, including those not quantified in this study. Future work should seek to directly evaluate the impact of CW design changes in patients with DFU and the relationship to adherence.

## 1. Introduction

Up to 34% of persons with diabetes will experience a diabetic foot ulcer (DFU) within their lifetime [1], and up to 15% of these DFUs may never heal [2], with many non-healing DFUs requiring amputation [2]. DFUs typically form in response to repetitive cycles of physical stress induced by weight-bearing activity. Once formed, continued physical stress may inhibit healing. Thus, a central tenet to heal DFUs is “offloading” areas of high pressure on the plantar surface of the feet via specialized footwear [3]. While the irremovable total contact cast (TCC) remains a first-line choice for offloading, only 1.7% of US clinics use the TCC in treating the majority of their cases [4] and the US Wound Registry concluded that “total contact casting is vastly underutilized in DFU wound care settings” [5]. Current guidelines from the International Working Group on the Diabetic Foot (IWGDF) recommend traditional TCC or knee-high cast walkers rendered irremovable as equivalent first-line choices, and recommend either knee-high or ankle-high offloading devices as the next best options [6]. Although clinicians commonly prescribe removable knee-high and ankle-high cast walkers to offload DFUs [4,7], these devices can promote unstable and asymmetric gait patterns by inducing a limb length discrepancy (LLD) and limiting ankle motion. Such gait patterns, may increase the metabolic costs of walking with a CW, which is not trivial for patients with DFUs given that they are more highly fatigable [8] and have a lower tolerance for increased metabolic demands [9,10]. However, the effect of CW design on metabolic costs of walking and CW use is not well studied.

CWs typically include a thick rocker bottom sole to facilitate offloading, which induces an LLD that can promote compensatory behaviors and increase the metabolic costs of walking. Indeed, inducing only a 2 cm LLD with an artificial lift results in greater oxygen consumption and higher ratings of perceived exertion than walking with no LLD [11]. In response to an imposed LLD, individuals walk with greater pelvic obliquity (drop) toward the shorter limb [12] to reduce the effective length of the longer limb. This significantly alters the lateral motion of the center of mass (CoM) and related stability [13,14]. Specifically, compared to normal gait, there is greater peak lateral excursion of the CoM toward the stance limb when the shorter limb is in stance and less when the longer one is. To counter changes in stability resulting from altered lateral CoM motion, individuals may adopt a wider step when walking with an LLD [15]. While wider steps can assist with redirecting the lateral motion of the CoM during step-to-step-transitions (double support), this is at the expense of greater energy expenditure; metabolic costs increase disproportionately with increasing step width [16]. Increased pelvic drop, and associated changes to CoM displacement, can also foster temporal changes to the gait pattern such as increased step time on the short limb and decreased on the long, resulting in a significant between-limb asymmetry [15,17]. Whereas healthy, normal gait is characterized by spatiotemporal symmetry, deviations from symmetry, particularly in step time, are associated with increased metabolic costs [18,19]. These increased costs reflect an increase in mechanical power to compensate for altered pendular (CoM) dynamics resulting from asymmetry. Accordingly, provision of a contralateral lift to counter induced LLD occurring from use of a CW may foster narrower steps with more symmetric timing, lowering metabolic costs compared to a CW alone.

Asymmetries in step length can also significantly alter the metabolic demand of walking [19]. While LLD alone can contribute to moderate asymmetries in step length [17], ankle immobilization with CW use [20,21] may play a more substantial role in generating these asymmetries. By immobilizing the ankle, CWs limit the generation of propulsive forces [21–23], which can promote shorter relative steps with the limb contralateral to the CW, i.e., when the limb with the CW is involved in push off. The reduced ability to generate positive mechanical work with a CW also disrupts the exchange of positive and negative work during step-to-step transitions forcing muscles to generate greater positive work at other joints to redirect the CoM [21–23]. This increase in step-to-step transition costs manifests as greater metabolic costs and has been implicated as a mechanism for the greater metabolic costs of walking in patient populations with unilateral deficits in push off force generation, e.g., lower limb prosthesis users [24]. Accordingly, CW design modifications that allow for greater ankle range of motion (capacity for force generation) may help to produce a more symmetric, economical gait. A recent systematic review of kinematic changes with the use of CWs suggest that ankle-high CWs, i.e., those designed with struts or encasements that stop just above the ankle, significantly increase the ankle range of motion compared to knee-high walkers, i.e., those with struts that run the entire length of the shank [25]. The lower cut of the ankle-high CW also reduces the weight of the CW by ~20% [26], which may help to counter increased metabolic costs associated with adding weight at the ankle [22,27]. Importantly, while knee-high removable CWs were historically the most strongly recommended removable offloading device [28], the most recent guidelines from IWGDF suggest using either a knee-high or an ankle-high CW as the first option for removable offloading [6].

The purpose of this study was to evaluate the effects of different commercially available removable cast walker options on the metabolic costs and perceived exertion of walking, and on related spatiotemporal gait kinematics, in healthy young participants as an initial step to understanding factors that may impact CW adherence in patients with DFUs. Specifically, we sought to evaluate the effects of reducing the height of the CW struts and of providing a contralateral shoe lift to limit induced LLDs. We hypothesized that H1) regardless of CW design, the metabolic costs and perceived exertion of walking would be higher when using a CW than when using standard athletic footwear; H2) both strut height and the use of a lift would significantly alter the metabolic costs and perceived exertion of walking, with lower values for the ankle-high CW vs. a knee-high CW and for the lift vs. no lift condition; H3a) step width and step time asymmetry would be lower when using a lift, regardless of strut height; and H3b) step length asymmetry would be lower in an ankle-high vs. knee-high CW. Support of H3 would help to explain changes in metabolic costs across CW conditions.

## 2. Materials and Methods

A convenience sample of fourteen adults were recruited under the Institutional Review Board-approved study. No formal a priori power analysis was performed, as this study was essentially a “proof-of-concept” in a homogeneous healthy sample, undertaken as an initial step to understanding CW usability in patients with DFUs. Nonetheless, samples of ~12 participants have been recommended to estimate variances and assess proof of concept [29,30]. Thus, our convenience sample of 14 healthy volunteers is adequate to demonstrate the presence (or absence) of the expected changes. All participants were free of neurodegenerative or musculoskeletal diseases that interfered with gait or balance, physical or respiratory disabilities, and lower extremity injuries.

Before data collection, the Oxycon Mobile (Vyaire Medical, Mettawa, IL, USA) volume and gas analyzers were calibrated to the ambient conditions. Study participants were then fit with a silicone mask worn over the mouth and nose. Suction was tested to ensure proper fit. The mobile unit captured volumetric oxygen consumption (VO_2_) on a breath-by-breath basis during all tasks.

At the start of the data collection, the participant sat in a chair until VO_2_/kg reached 3–4 mL/kg·min (resting levels). The participant then stood for two minutes on a single-belt motorized treadmill with an embedded force plate for quantifying spatiotemporal gait parameters (C-Mill; Motek Medical; Amsterdam, The Netherlands), while wearing laboratory-provided standardized athletic footwear. Thereafter, the participant walked for 6 min in the same shoes. Walking speed was normalized to the participant’s leg length (*l*) according to the following: $\text{speed}=\sqrt{F_{n}\cdot g\cdot l}$, where *g* is the gravitational constant and *F_n_* is the Froude number, set to 0.20 [31].

**Figure 1.**
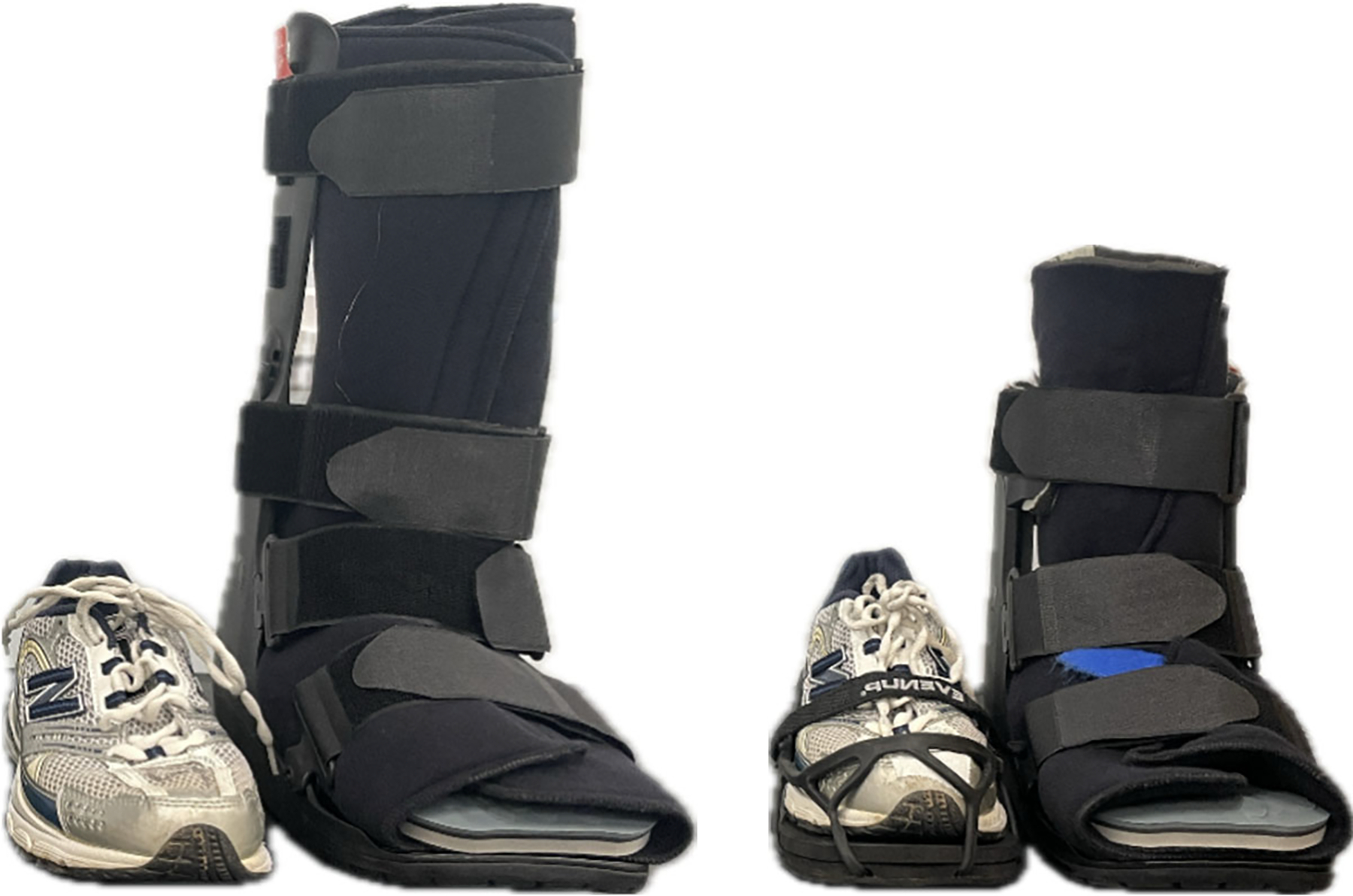
Two of the four CW conditions evaluated. The left figure demonstrates the knee-high CW with no contralateral lift (i.e., standard footwear). The right figure demonstrates the ankle-high CW with a contralateral lift. The remaining two CW conditions (not shown) represent combinations of these.

After completing the standing and walking trials with athletic shoes, the participant was fit to the first of four CW conditions (Figure 1), which included (1) knee-high CW (DH Walker; Ossur, Reyjavik, Iceland) without contralateral lift; (2) knee-high CW with contralateral lift (EVENup, OPED Medical Inc., Braselton, GA, USA); (3) ankle-high CW (DH Walker insole in tandem with a low top Equalizer Walker; Ossur, Reykjavik, Iceland) without contralateral lift; and (4) ankle-high CW with contralateral lift. The order of the conditions was randomized. In all CW conditions, the standardized athletic footwear was worn on the contralateral foot. Once a CW was fit for a condition, participants walked around the lab until they reported that walking felt “as natural as possible” (minimum of 1–2 min). The participant then sat in a chair until their VO_2_/kg returned to resting level, stood on the treadmill for 2 min, and walked at the normalized speed for 6 min.

Between each condition, participants were asked about their perceived exertion using the Borg Rating of Perceived Exertion (RPE) scale. Using this scale, people rated the intensity of physical activity from 6, indicating no perceived exertion at all, to 20, indicating maximal exertion [32]. RPE data from one subject was not available for analysis.

For each standing and walking condition, we averaged the rate of oxygen consumption during two minutes of the trials (i.e., the entire standing trial or the last two minutes of the walking trial). The rate of metabolic energy expenditure (RMEE), in units ml O_2_/min/kg, was determined by normalizing this value by body mass. Finally, the metabolic cost of transport (MCoT) was calculated as the difference in the RMEE for walking and standing normalized by walking speed and expressed in units of ml O_2_/kg/m. The lower the cost, the more economical the gait, i.e., less oxygen consumption per kg to move the center of mass over a given distance.

The mean step length (SL) on each limb and a single value for the mean step width across both limbs (SW) were provided directly by the C-Mill treadmill. The treadmill also provided timing of gait events (heel strike and toe off) for each limb, which were entered into a custom code (Matlab, Mathworks, Cambridge, MA, USA) to calculate the step time (ST) for each step and the associated mean values. Asymmetries in SL and ST were calculated using the mean values and a symmetry index (SI):

$$\text{SI}=\left|\frac{\text{Left}-\text{Right}}{0.5*(\text{Left}+\text{Right})}\right|*100\%$$

This is consistent with the SI proposed by Robinson et al. [33]. Here, we included the absolute value as any asymmetry, even if in the direction opposite of expectation, should help to explain the metabolic costs of walking.

Linear mixed models (LMMs) were used to test all hypotheses, accounting for missing data (for two subjects the treadmill force plate did not properly function for some conditions, and for one participant the RPE was not taken for all conditions). We first ran an LMM for all outcomes (MCoT, RPE, SW, SL SI, and ST SI), including a 5-level fixed (repeated) factor for footwear condition and a random intercept for subjects. Planned comparisons were performed using a Least Square Difference correction to compare CW conditions to the control, and normality of residuals was verified by visual inspection of histograms and Q-Q plots. To directly compare CW conditions for H2 and H3, we ran LMM that included three fixed factors: strut height (ankle-high vs. knee-high), lift use (yes/no) and their interaction. The model also included a random intercept for subjects. In the event of a significant interaction, we performed post-hoc pair-wise comparisons using a Least Square Difference correction to determine which specific conditions differed. The normality of residuals was verified by visual inspection of histograms and Q-Q plots. All analyses were performed in IBM SPSS 20 (IBM, Armonk, NY, USA) with significance set at the level of *p* ≤ 0.05.

## 3. Results

The 14 recruited participants represented a homogenous, young cohort (age: 25.71 ± 2.87 years, height: 1.70 ± 0.11 m, mass: 69.32 ± 15.15 kg).

### 3.1. Metabolic Data

A one-factor LMM revealed a significant effect of condition on MCoT (F(4,52) = 13.46, *p* < 0.001). Individual contrasts indicated that MCoT was significantly higher in all four CW conditions compared to the control condition (all *p* < 0.001). Specifically, MCoT in the control condition was 0.0103 ± 0.010 mL/kg·m, while the mean MCoT values in the CW conditions ranged from 0.116 to 0.122 mL/kg·m (see Table 1 for details).

**Figure 2.**
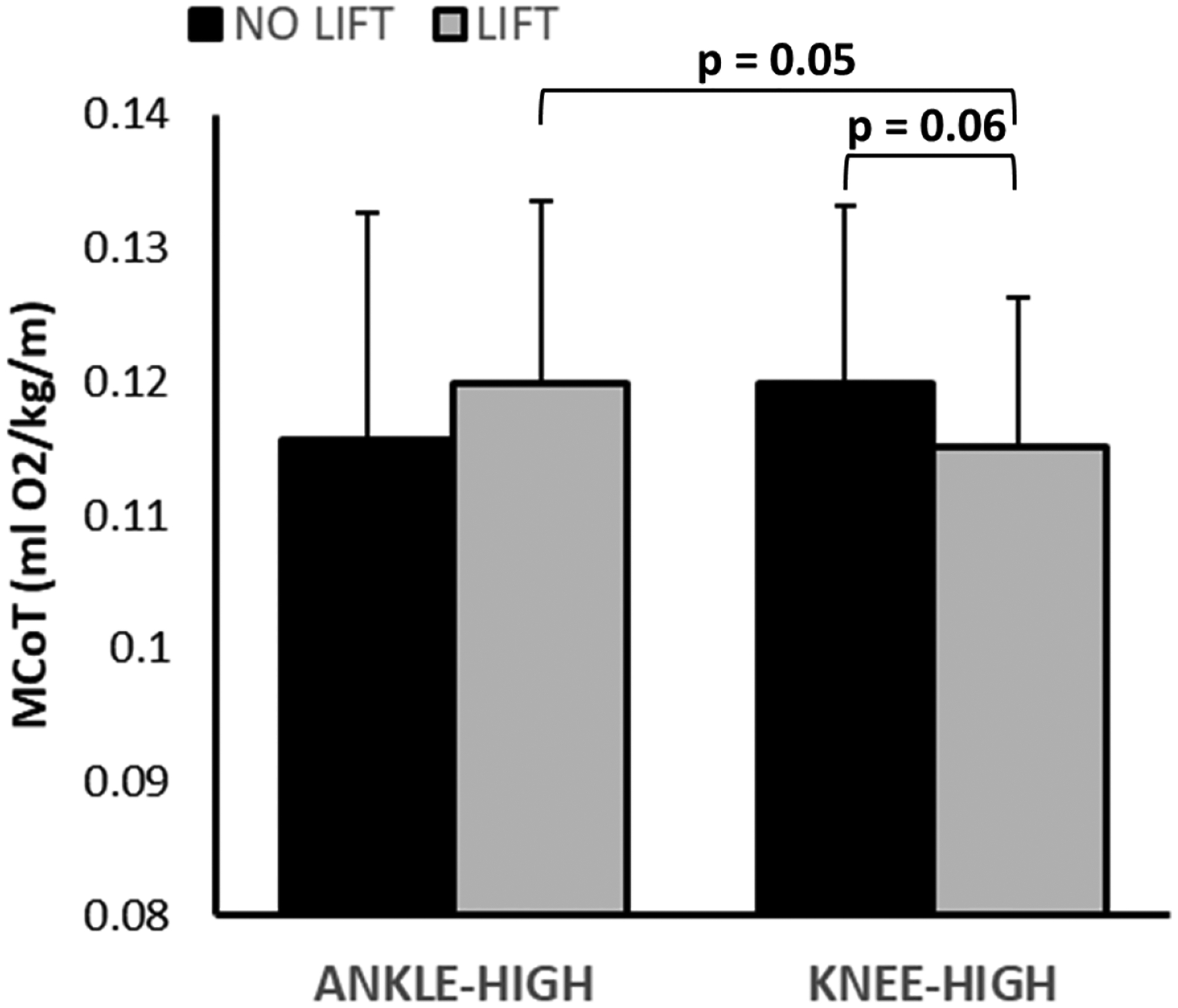
Metabolic cost of transport (MCoT) for each of the four CW conditions. There was no significant effect of strut height (left two bars vs. right two bars) or contralateral lift (dark bara vs. light bars) on MCoT. However, a significant (*p* = 0.021) interaction was observed with the contrasts most strongly contributing to that effect indicated.

The three-factor LMM revealed a significant interaction (F(1,39) = 5.76, *p* = 0.021). Post-hoc comparisons revealed two borderline significant effects. When using a lift, ankle-high struts resulted in a slightly higher MCoT compared to knee-high struts (F(1,39) = 4.02, *p* = 0.052). Additionally, using a lift in conjunction with a knee-high CW was associated with slightly lower MCoT compared to using no lift (F(1,39) = 3.89, *p* = 0.056) (see Figure 2).

### 3.2. RPE

A one-factor LMM revealed a significant effect of condition on RPE (F(4,48) = 21.20, *p* < 0.001). Evaluating contrasts indicated that RPE was significantly higher in all four CW conditions compared to the control condition (all *p* < 0.004). Specifically, RPE in the control condition was 8.84 ± 1.21, while the mean RPE values in CW conditions ranged from 9.77 to 11.46 (Table 1).

**Figure 3.**
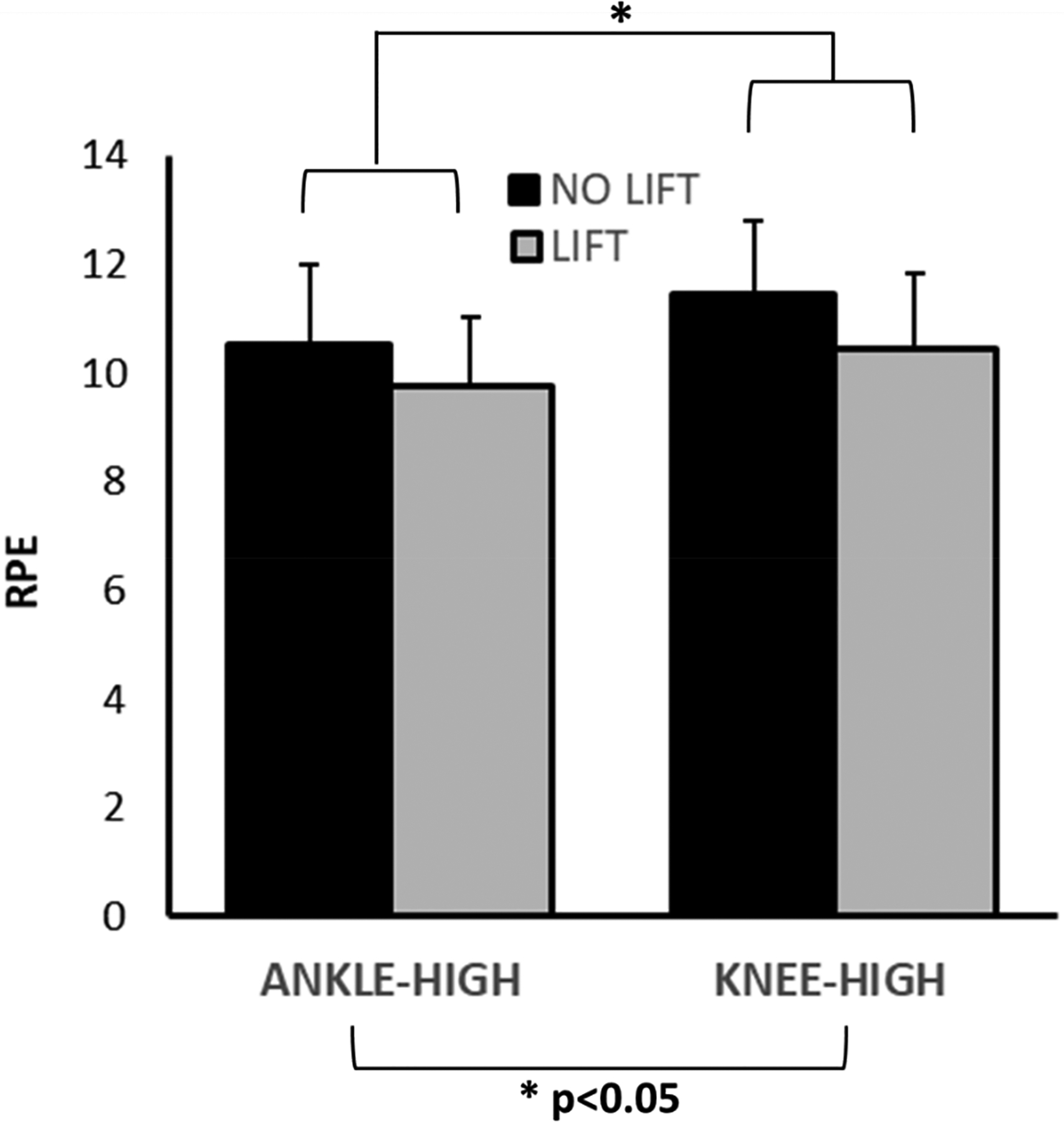
Rating of Perceived Effort (RPE) across each of the four CW conditions. There was a significant main effect of strut height (left two bars vs. right two bars) and of contralateral lift (dark bars vs. light bars) on RPE, with higher struts and a larger induced LLD (no lift) promoting greater perceived effort of walking.

The three-factor LMM revealed significant effects for strut height (F(1,36) = 16.13, *p* < 0.001) and for lift (F(1,36) = 19.35, *p* < 0.001) but no significant interaction (F(1,36) = 0.33, *p* = 0.570). RPE was greater in the knee-high CW compared to the ankle-high CW (estimated marginal means ± standard error—10.96 ± 0.35 vs. 10.15 ± 0.35, respectively; Figure 3) and without a lift compared to with a lift (11.00 ± 0.35 vs. 10.11 ± 0.35, respectively).

### 3.3. Spatiotemporal Kinematics

**Figure 4.**
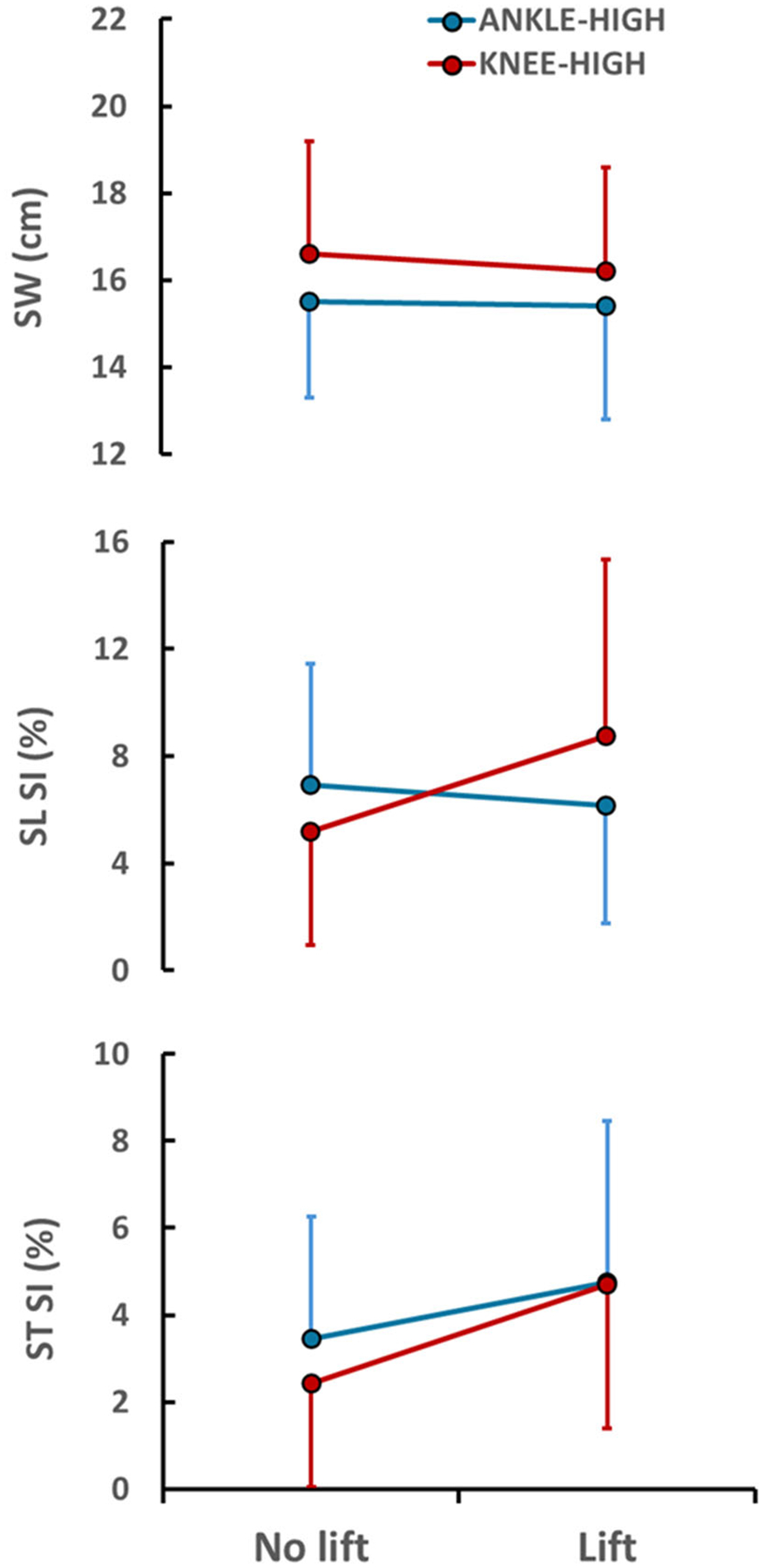
Spatiotemporal kinematics across each of the four CW conditions. (**top**) Step width (SW) in the knee-high CW (red) was significantly greater than with the ankle-high CW (blue). (**middle**) There was a significant height × lift interaction for symmetry index (SI) for step length (SL), with post hoc comparisons indicating the SI was lower for the knee-high CW compared to the ankle-high CW only when using a lift (left two data points; *p* = 0.08); SI SL was more similar for the two when using a lift (right two points; *p* = 0.22). (**bottom**) Counter to expectations, regardless of strut height, the SI for step time (ST) was higher when using a lift compared to no lift.

We did not observe a systematic effect of footwear condition on spatiotemporal kinematics, with the effects varying by measure (Table 1 and Figure 4). There was a main effect of condition on SW (F(4,49.5) = 12.26, *p* < 0.001) with CW conditions having SWs that were 2.9–4.1 cm wider than the control (*p* < 0.001 for all). There was also a main effect of condition on SL SI (F(4,49.5) = 4.18, *p* = 0.005) with SI greater than control for all except the knee-high CW with no lift (*p* < 0.036 for significant comparisons; *p* = 0.136 for knee-high without lift). Finally for ST SI, the overall effect for footwear condition did not reach significance (F(1,49.5) = 2.26, *p* = 0.076).

When comparing kinematics across CW conditions, we again observed non-systematic effects. For SW, there was a main effect of CW height (F(1,37) = 4.67, *p* = 0.037), with a 7% increase for knee-high relative to ankle-high (*p* > 0.62 for lift effect and height × lift interaction). For SL SI, there was a significant height × lift interaction (F(1,37) = 4.34, *p* = 0.044). Post-hoc tests revealed a significant, 3.5 point reduction in SL SI using a knee-high CW with vs. without a lift (*p* = 0.026); when using an ankle-high CW there was no significant effect of a contralateral lift on SL SI (*p* = 0.51). Within a given lift condition, there were no differences in SL SI between strut heights (all *p* > 0.12). For ST SI, there was a significant effect of lift (F(1,38) = 7.14, *p* = 0.011), with an unexpected increase of 1.8 points in ST SI when using a lift (*p* = 0.011); there was no significant effect of CW strut height, nor a significant interaction of height × lift for ST SI (*p* > 0.42 both).

## 4. Discussion

The purpose of this study was to evaluate the effects of different commercially available cast walker options on the metabolic costs and perceived exertion of walking, and on related spatiotemporal gait kinematics, in healthy young participants as an initial step to understanding CW usability in patients with DFUs. We hypothesized that regardless of CW design, the metabolic costs and perceived exertion of walking would be higher when using a CW than while wearing standard athletic footwear. This hypothesis was fully supported. All four CW conditions showed higher RPE and MCoT compared to the control condition (Table 1). Secondly, we hypothesized that the metabolic requirements and perceived exertion of walking would be lower with an ankle-high CW vs. a knee-high CW and when using a CW with a contralateral shoe lift compared to without a lift. This hypothesis was only partially supported. While there was a significant main effect of lift and height on RPE (Figure 3), these effects were absent for MCoT (Figure 2), although the use of a lift did reduce MCoT for the knee-high CW. This suggests that the perception of exertion is not a direct reflection of metabolic costs. Third, we hypothesized that SW and ST SI would be lower when using a lift, regardless of strut height. This hypothesis was not supported. In fact, the use of lift resulted in greater ST SI and no change in SW (Figure 4). Nonetheless, differences in ST SI between CW conditions may have little implications for behavioral changes with CW use given the absence of significant differences between any CW condition and the control condition. Although SW was not significantly altered by the use of a lift, there was a significant effect of strut height on SW, which may relate to greater ankle immobilization in the knee-high CW [25]. CWs not only limit sagittal plane ankle range of motion (RoM) but frontal plane motion, as well [21]. If frontal plane ankle RoM is more compromised in a knee-high than an ankle-high CW, as is the case for sagittal plane motion [21,25], then mediolateral walking stability would be more affected; to compensate, participants would need to take wider steps in the knee-high CW. We further hypothesized that SL SI would be lower in an ankle-high vs. knee-high CW, which was not supported. In fact, SL SI was significantly greater for the ankle-high vs. knee-high CW, at least in the case with no lift, which is not easily explained. Still, the fact that both MCoT and gait kinematics behaved unexpectedly provides a degree of internal consistency whereby the latter explains the former.

The unexpected kinematic changes and their minimal effect on the metabolic cost of transport (MCoT) suggest a complex interplay between gait variables. These findings indicate that changes in one gait parameter that are expected to increase MCoT may be offset by compensatory adjustments in other variables that reduce it, maintaining walking economy; or that concurrent changes in variables that are expected to increase MCoT may not be additive. This possibility is supported by a study from Stenum and Cho, which demonstrated that while asymmetries in step length and step time each independently contributed to metabolic costs, when both are present metabolic power is primarily driven by asymmetries in step time [19]. Thus, gait variables can interact non-linearly to impact metabolic costs. Another example of this complex interaction is the theoretical relationship between SW and ST governed by pendular dynamics. As ST increases, the body has more time to fall toward the stance limb, which promotes greater lateral excursion of the center of mass [34]. This, in turn, often necessitates a wider step width to maintain stability. However, our findings show that step width can also be modified independently of step time, evidenced by an effect of strut height on SW but not ST SI (Figure 4). Furthermore, small changes in step time that may not be large enough to significantly influence step width or MCoT can be amplified in step time asymmetry, as equal and opposite changes in limb timing result in a two-fold change in the symmetry index. Unexpected kinematic changes that minimally impact MCoT may also represent a behavioral choice by participants to intentionally adjust their gait to avoid excessive energetic costs across conditions. Indeed, while humans generally prefer to walk symmetrically, under certain conditions they may be willing to walk asymmetrically if it reduces metabolic costs [35].

While expectations regarding MCoT were not supported, those regarding perceived exertion were, which may have important implications for the long-term goal of the work, i.e., improving the usability of CWs to positively impact user’s behaviors. While the Borg RPE scale has been shown to associate with physiological indicators of exercise intensity [36], the two are not necessarily causally linked. A number of studies have demonstrated disassociations between metabolic stressors and perceived effort [37]. This may, in part, reflect the fact that perceived exertion represents the combined effects of muscular or joint strain, sensations from the cardiorespiratory system, and the internal awareness of the generation of central commands on these systems [38]. In this regard, RPE may be more sensitive than MCoT for detecting the collective impact of the complex, interacting biomechanical changes that may occur with the provision of different CW designs. While dissociations between perceived and physiological exertion are often shown for more intense activities than walking at a comfortable speed (e.g., cycling, running, resistive training), a study by Caldwell et al. well demonstrates a disassociation during locomotion [39]. In that study, healthy adults walked naturally and then adopted strategies with reduced knee joint loading, such as “toeing out” (i.e., walking with externally rotated feet). Walking with these strategies led to a trivial (2%) increase in the metabolic cost of walking but a more than three-fold increase in perceived exertion [39].

Despite reduced weight of the ankle-high vs. knee-high CW, walking in an ankle-high CW was not more economical. While manufacturer specifications indicate the ankle-high CW is approximately 200 g lighter than the knee-high CW, in healthy young subjects, oxygen consumption increases by only 1% for every 100 g of mass added at the ankle [27]. We are likely underpowered to detect the 2% change in MCoT associated with the added mass. Previous studies of healthy adults comparing designs of firefighter boots included samples of ~25 people to observe significant changes in metabolics when weight differences were five times greater (~1 kg) [40,41]. The distribution of the added mass may also explain why MCoT did not increase with the heavier, ankle-high CW. The additional mass of the knee-high CW is distributed around the proximal portion of the leg. Placing mass more proximally, at least when added to transtibial prostheses, reduces the impact of the added mass on the energetic costs of walking [42]. Finally, the study design may have contributed to non-varying MCoT across conditions. Although self-selected walking speed reduces with CW use [25], we opted to standardize walking speed across footwear conditions to eliminate speed as a confounding variable. However, given that the cost of transport varies with walking speed [43], if study participants were allowed to select different walking speeds across footwear conditions, those differences could have translated to differing MCoT. Indeed, acute responses of CWs have been shown to differ when speed is standardized compared to when speed is freely chosen [23].

Although prior work indicates that artificially inducing an LLD, at least in older adults, significantly increases net oxygen consumption relative to walking without the LLD [11], we did not observe higher MCoT in the presence of an induced LLD (i.e., no main effect of lift). This could reflect an insufficiently large enough LLD to observe effects, as the literature suggests differences of 2–3 cm are needed to observe measurable changes in gait economy [11]. Given that the lift was set to the maximum allowable measure of 2.5 cm, we assume that the LLD was at least of a sufficient magnitude to observe change. However, this may still have been insufficient to fully compensate for the LLD, leading to higher-than-normal MCoT persisting even with the provision of the lift, at least in the ankle-high device. On the other hand, the absence of an effect agrees with observations that younger adults with real LLD walk with smoother, more economical gait patterns compared to those without an LLD [44]. Young adults are capable of developing beneficial compensatory strategies, at least in response to habitual experience.

Given that patients with DFUs were not evaluated, it would be premature to provide specific recommendations regarding prescription practices for this population, although some clinical implications are worth noting. It is well documented that patients with DFU have low adherence related to CW use, often wearing the device for <60% of their weight-bearing activities [45,46]. Factors that negatively impact user experiences with the device, such as high metabolic costs, and/or high perceived exertion may negatively impact behaviors, with the latter playing a critical role for patients with diabetes, who perceive harder effort for the same amount of work [47]. Indeed, in older adults with mobility limitations, perceived effort of walking is strongly associated with physical activity levels [48], and perceived exertion may be “a more important determinant of activity limitation than metabolic energy expenditure” [49]. Perceptions of CW heaviness may also be critical for determining behavior given that perceived heaviness can predict adherence [50], even if the device’s mass does not significantly impact MCoT. Moreover, the lighter ankle-high CW may improve stability and enhance walking ability and mobility relative to the knee-high option [26,50,51]. Concurrent use of a lift may further enhance this, providing a level of stability (and of comfort) which exceeds that of a knee-high CW [51]. Improvement in stability is not trivial as self-reported postural instability is a powerful predictor of DFU patients’ non-adherence with a CW [45]. Overall, even if the ankle-high CW and/or lift do not lower MCoT, a number of other benefits inherent to the combination may contribute to improved adherence. Any small reductions in offloading that might occur when using a CW with lower struts [26,51] are likely more than made up for by higher adherence, i.e., avoidance of excessively high loads inherent to walking without a CW. We suggest that prescribers’ decisions should not solely be based on the functionality (offloading capacity) of the device. Providers should also consider how the patient will interact with the device. Future studies should focus on the effects of strut height and lift on MCoT and RPE in patient populations, including more comprehensive biomechanical evaluations (e.g., joint kinetics and muscle activation patterns) to fully explain metabolic demands, and longitudinal evaluation of adherence, to determine the extent to which MCoT and RPE predict CW use. Collectively, this information will help to justify the use of specific offloading devices, with a focus on the impact of these device features on patient adherence.

There are several limitations of the current work. First, the study included only healthy young subjects, in whom adherence is unlikely to be an issue, as an initial step in relating changes in metabolic costs to adherence in patients with DFUs. Young subjects primarily wear CWs to manage offloading in response to trauma and/or surgery, both of which would be expected to cause pain if the user were to weight-bear without the CW. In contrast, patients with DFUs generally present with comorbid peripheral neuropathy, which removes any negative feedback (i.e., pain) if the patient chooses to weight-bear without the device. The extent to which findings from the current study extrapolate to patients with DFUs remains to be determined. A second limitation is that we assessed only immediate changes in metabolic costs after providing CW devices. Patients are often prescribed CWs for extended periods over which they may develop compensatory strategies to improve metabolic economy [52]. The extent to which the results would persist and impact usability across the duration of CW usage is unknown. Finally, this study is limited in its ability to provide a comprehensive mechanism explaining changes, or lack thereof, in MCoT across CW conditions. Indeed, biomechanical compensations in response to inducing an LLD [53], and in response to wearing a CW [25] can be numerous, and only a few variables were measured herein. Despite these limitations, to the best of our knowledge, this is the first study to evaluate metabolic changes when manipulating LLD in the presence of a CW, whereas previous related studies have tended to focus only on induced LLDs using a lift on one limb, with few directly evaluating metabolic costs in orthopedic devices.

## 5. Conclusions

The current study aimed to investigate the impact of different designs of cast walkers (CWs) on metabolic costs and perceived exertion of walking, with the long-term goal of improving adherence to offloading interventions. Despite expectations, we observed no significant effect of CW strut height or of the presence of the lift on MCoT. Similarly, changes in spatiotemporal gait parameters were not aligned with expectations. While the reason for unexpected biomechanical changes is unclear, they are consistent with the absence of main effects for MCoT. Although metabolic costs were not significantly affected by CW conditions, perceived exertion was significantly reduced with use of an ankle-high CW and with provision of a contralateral lift. The fact that RPE but not MCoT demonstrates effects suggests independence in their information, with RPE potentially more sensitive to the collective changes in behavior across CW conditions. Future studies should focus on the effects of strut height and lift on MCoT and RPE in patient populations, including more comprehensive biomechanical evaluations, with a focus on the impact of these features on patient adherence, to help justify the use of specific offloading devices.

## Figures and Tables

**Table 1.** Summary of metabolic cost and perceived exertion of walking by condition.

Outcome	Condition					*p*-Values				
	Control	Knee-High		Ankle-High		vs. Control	Height	Lift	Lift × Height	
		Lift	No Lift	Lift	No Lift					
**MCoT (mL/m/kg)**	0.013 ± 0.010	0.116 ± 0.010	0.122 ± 0.014	0.122 ± 0.012	0.117 ± 0.012	**KHL: <0.001** **KHNL: <0.001** **AHL: <0.001** **AHNL: <0.001**	0.66	0.70	0.02	NL-KH vs. AH: 0.17 L-KH vs. AH: 0.05 AH-L vs. NL: 0.16 KH–L vs. NL: 0.06
**RPE**	8.85 ± 1.21	10.46 ± 1.39	11.46 ± 1.33	9.77 ± 1.24	10.54 ± 1.45	**KHL: <0.001** **KHNL: <0.001** **AHL: 0.003** **AHNL: <0.001**	**<0.001**	**<0.001**	0.57	--
**SW (cm)**	12.49 ± 1.06	16.16 ± 2.36	16.56 ± 2.58	15.40 ± 2.17	15.48 ± 2.55	**KHL: <0.001** **KHNL: <0.001** **AHL: <0.001** **AHNL: <0.001**	**0.03**	0.63	0.70	--
**SL SI**	2.76 ± 1.33	8.76 ± 6.56	5.18 ± 4.22	6.14 ± 4.37	6.92 ± 4.54	**KHL: <0.001** KHNL: 0.14 **AHL: 0.04** **AHNL: 0.008**	0.88	0.27	**0.04**	NL-KH vs. AH: 0.08 L-KH vs. AH: 0.22 AH-L vs. NL: 0.94 KH–L vs. NL: 0.053
**ST SI**	2.77 ± 3.15	4.71 ± 3.32	2.43 ± 2.39	4.74 ± 3.71	3.44 ± 2.81	KHL: 0.08 KHNL: 0.69 AHL: 0.07 AHNL: 0.57	0.42	**0.01**	0.50	--

Note: Data presented as mean ± standard deviation. MCoT—metabolic cost of transport; KHL—knee-high with lift; KHNL—knee-heigh with no lift; AHL—ankle-high with lift; AHNL—ankle-high with no lift; bold values represent *p* ≤ 0.05.

## Data Availability

The raw data supporting the conclusions of this article will be made available by the authors on request.

## Abbreviations

The following abbreviations are used in this manuscript:

CW: cast walker
DFU: diabetic foot ulcer
LLD: limb length discrepancy
MCoT: metabolic cost of transport (MCoT)
RMEE: rate of metabolic energy expenditure
RPE: rating of perceived exertion (using the Borg scale)
VO_2_: volumetric oxygen consumption
